# Impact of Geriatric Hotlines on Health Care Pathways and Health Status in Patients Aged 75 Years and Older: Protocol for a French Multicenter Observational Study

**DOI:** 10.2196/15423

**Published:** 2020-02-13

**Authors:** Laure Martinez, Noémie Lacour, Régis Gonthier, Marc Bonnefoy, Luc Goethals, Cedric Annweiler, Nathalie Salles, Nathalie Jomard, Jérôme Bohatier, Magali Tardy, Etienne Ojardias, Romain Jugand, Bienvenu Bongué, Thomas Celarier

**Affiliations:** 1 Department of Clinical Gerontology, University Hospital of Saint-Etienne Saint Etienne France; 2 Department of Clinical Gerontology, Firminy Hospital Firminy France; 3 Department of Clinical Gerontology, University Hospital of Lyon Sud Lyon France; 4 Jean Monnet University Saint-Etienne, Chaire Santé des Ainés Saint Etienne France; 5 Support and Education Technic Centre of Health Examination Centres (CETAF) Saint Etienne France; 6 Department of Clinical Gerontology, Angers University Hospital; Angers University Memory Clinic, Research Center on Autonomy and Longevity, University of Angers Angers France; 7 School of Medicine, Health Faculty, University of Angers Angers France; 8 Robarts Research Institute, Department of Medical Biophysics, Schulich School of Medicine and Dentistry, University of Western Ontario London, ON Canada; 9 Department of Clincal Gerontology, Bordeaux University Hospital Pessace France; 10 Department of Clinical Gerontology, Clermont-Ferrand University Hospital Clermont-Ferrand France; 11 Department of Clinical Gerontology, Saint Chamond Hospital Saint Chamond France; 12 Jean Monnet University Saint-Etienne Autonomic Nervous System Research Laboratory, University of Lyon Saint Etienne France; 13 Gerontopôle AURA Saint Etienne France

**Keywords:** aged, health care, hotline, emergency department, general practice medicine

## Abstract

**Background:**

In France, emergency departments (EDs) are the fastest and most common means for general practitioners (GPs) to cope with the complex issues presented by elderly patients with multiple conditions. EDs are overburdened, and studies show that being treated in EDs can have a damaging effect on the health of elderly patients. Outpatient care or planned hospitalizations are possible solutions if appropriate geriatric medical advice is provided. In 2013, France’s regional health authorities proposed creating direct telephone helplines, “geriatric hotlines,” staffed by geriatric specialists to encourage interactions between GP clinics and hospitals. These hotlines are designed to improve health care pathways and the health status of the elderly.

**Objective:**

This study aims to describe the health care pathways and health status of patients aged 75 years and older hospitalized in short-stay geriatric wards following referral from a geriatric hotline.

**Methods:**

The study will be conducted over 24 months in seven French university hospital centers. It will include all patients aged 75 and older, living in their own homes or nursing homes, who are admitted to short-stay geriatric wards following hotline consultation. Two questionnaires will be filled out by medical staff at specific time points: (1) after conducting the telephone consultation and (2) on admitting the patient to a short-stay geriatric medical care. The primary endpoint will be mean hospitalization duration. The secondary endpoints will be intrahospital mortality rate, the characteristics of patients admitted via the hotline, and the types of questions asked and responses given via the hotline.

**Results:**

The study was funded by the National School for Social Security Loire department (École Nationale Supérieure de Sécurité Sociale) and the Conference for funders of prevention of autonomy loss for the elderly of the Loire department in November 2017. Institutional review board approval was obtained in April 2018. Data collection started in May 2018; the planned end date for data collection is May 2020. Data analysis will take place in the summer of 2020, and the first results are expected to be published in late 2020.

**Conclusions:**

The results will reveal whether geriatric hotlines provide the most effective management of elderly patients, as indicated by shorter mean hospitalization durations. Shorter hospital durations could lead to a reduced risk of complications—geriatric syndromes—and the domino chain of geriatric conditions that follow. We will also describe different geriatric hotlines from different cities and compare how they function to improve the health care of the elderly and pave the way toward new advances, especially in the organization of the care path.

**Trial Registration:**

ClinicalTrials.gov NCT03959475; https://clinicaltrials.gov/ct2/show/NCT03959475

**International Registered Report Identifier (IRRID):**

DERR1-10.2196/15423

## Introduction

The world’s populations are aging, which represents a serious public health issue. There are many consequences categorized into four main types: (1) demographic, with increasing numbers and proportions of people older than 65 years with increased life expectancy; (2) epidemiologic, with an accumulation of chronic diseases, incapacitating conditions, and disabilities; (3) economic, with increased health costs and accelerated health care reforms; and (4) social.

The organizational model of hospitals is one of the first elements to be affected by population aging. Over time, the elderly will become the central focus of all hospitals. But how will these establishments address this issue? In France, emergency departments (EDs) are the fastest and most common means for general practitioners (GPs) to cope with the complex issues presented by elderly patients with multiple conditions [1,2]. These services are already overwhelmed, and studies show that being treated in EDs can have a damaging effect on the health of elderly patients [3]. These emergency services can be traumatizing for the elderly and cause the many different complications that are commonly grouped together as “geriatric syndromes” [4-10]. When treated in EDs, the elderly are at higher risk of functional decline due to falls, drug iatrogenesis, and incontinence [11-13].

For emergency physicians, treating elderly patients with multiple pathologies, who often arrive alone, proves to be challenging. Treatment is rendered even more difficult by the lack of time emergency physicians have at their disposal due to EDs being more and more overburdened.

Studies have reported that in 20% to 35% of cases, there was no real need for admission to EDs, and outpatient care or planned hospitalizations would have been possible if appropriate geriatric care was available [14]. Therefore, nearly a quarter of these hospitalizations could have been anticipated and prevented. Some studies on hospitalization durations report that patients with initially planned hospitalizations seem to experience longer times being seen and treated in EDs. Furthermore, this significant number of hospitalizations creates financial issues. According to the French High Council for the Future of Health Insurance, an estimated €2 billion are lost due to the segmentation and inadequacy of response, along with unnecessary hospitalizations [15]. Overall, the literature highlights the need to better coordinate outpatient care with hospital services to provide the elderly with access to more effective care [16].

To improve interactions between GP clinics and hospitals, France’s regional health authorities first organized the area as a unit focused on hospital centers incorporating both EDs and short-stay geriatric wards enabling immediate admission. In parallel, the regional health authorities created telephone hotlines designed to enhance GP clinic-hospital interactions with the aim of reducing the number of hospitalizations, particularly by improving the quality of response. The desired outcome was to improve both the care pathways and health status of the elderly. This hotline service was a recent creation (2013) and is still in an experimental form and not yet widespread. Its use still varies widely across the country, and we have yet to gauge its real value.

The primary objective of this study is to describe the health care pathways and status of patients aged 75 years and older hospitalized in short-stay geriatric care and referred via the hotline. The study population will be from different geriatric care hotlines from around France.

The aims of this study are to describe and compare how the geriatric medicine hotlines function with the goal of standardizing their use and defining ways they can be improved. We will also analyze the principal responses provided to GPs who call the hotlines and improve awareness of this tool. We hypothesize that geriatric hotlines will lead to reduced mean durations of hospitalizations by helping prevent time spent in EDs when it is unnecessary and potentially a risk. Through the use of a direct geriatric hotline diagnostic tool, we believe elderly patients will experience better health care pathways that no longer involve multiple trips to EDs. The hotlines may facilitate better adapted and more appropriate responses to situations that do not necessarily need hospitalization and provide better alternatives (eg, therapeutic medical and social care, outpatient care, consultations, ambulance services, telemedicine).

## Methods

### Design and Setting

**Figure 1:**
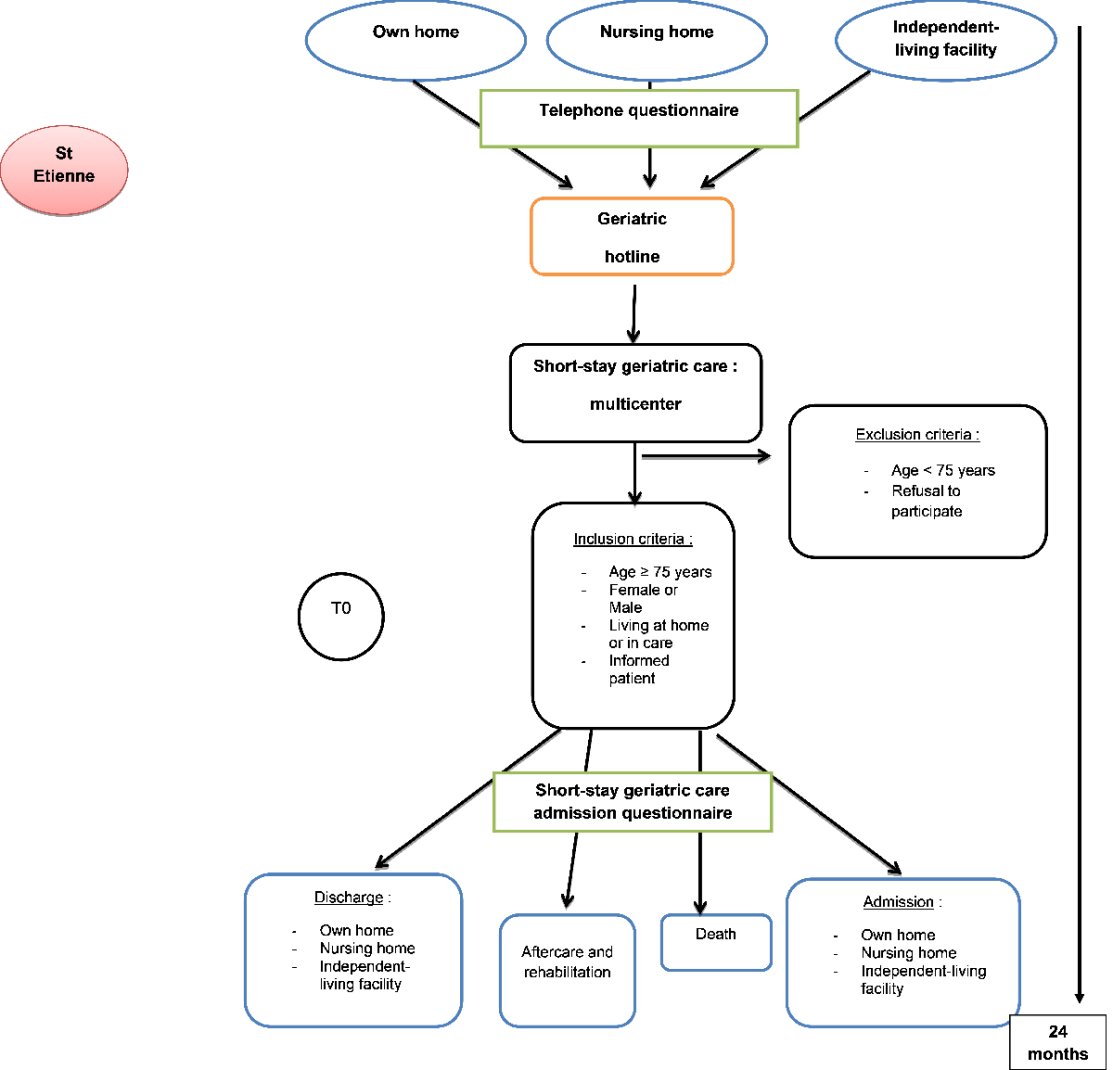
Flowchart of the study protocol.

This will be a multicenter descriptive study conducted in seven French investigating centers (including university centers, across three different regions) with subgroup analysis (Table 1). Participating establishments will all be voluntary. To participate in the study, the center must have a specific and separate telephone line for doctor-doctor communications. Only calls originating from health care professionals will be included. The study will last for 24 months (May 2018 to May 2020). The course of the study and its design are presented in Figure 1.

Two questionnaires will be distributed at two distinct time points: after telephone consultation (hotline) and after admission to a short-stay geriatric ward. The first questionnaire will be filled out via the hotline by the geriatric specialist answering the call. A second questionnaire will then be filled out during the patient’s hospitalization by the treating geriatric specialist.

**Table 1:** Description of geriatric care offered by different participating centers.

Paticipating center	Residents, n	Hospital beds, n	Short-stay geriatric care	Postdischarge care	Long-term rehabilitation care	Consultation	Outpatient clinic	Ambulance care	Telemedical consultation
Bordeaux	252,000	3076	x	x	x	x	x	x	x
Clermont-Ferrand	141,400	2136	x	x	x	x	x		
Saint Etienne	171,100	1819	x	x	x	x	x	x	x
Angers	151,500	1445	x	x	—^a^	x	x	x	x
Lyon Sud	513,300	1038	x	x	x	x	x	x	—
Saint Chamond	34,870	710	x	x	—	x	—	x	—
Firminy	16,840	475	x	x	x	x	x	x	—

^a^Service not offered.

### Participants

The study population will be people aged 75 years and older, living in their own home or a care facility (including independent-living facilities), hospitalized in short-stay geriatric care and referred by the hotline in one of the investigating centers. All participants will be fully aware of the study and their rights regarding their participation. All participating patients will give their consent. Participants or a legally authorized representative will receive informational brochures and give their informed oral consent (more visual difficulties and writing for the elderly). All participants will be able, at any moment and by any method, to withdraw from the study.

### Inclusion Procedure

When the patient is admitted to short-stay geriatric care, the investigators in each hospital of the study will first check the inclusion and noninclusion criteria of the patient. Then, they will explain the study to the patient and give the patient an information form. This will all be the responsibility of each center independently.

### Selection and Exclusion of Participants From Study

The inclusion criteria will be patients aged 75 years and older, male or female, living in their own home or a care facility (including independent-living facilities), hospitalized in short-stay geriatric care, and with confirmed receipt of information on the study and their rights, as stipulated by articles L.1122-1 of the French public health code, by 57 of the “Informatique et Libertés” data protection act, and by MR-003 of the French data protection and civil liberties authority (CNIL). The exclusion criteria will be patients aged younger than 75 years or refusing to participate.

### Monitoring

This study will involve no extra examinations or treatment besides traditional practices. The only difference will be the digitization of all medical findings for analysis. Data will be kept confidential and anonymized for analyses. This study will be noninterventional. All interventions will be carried out following normal clinical practices. There will be no unusual risk or specific constraint for participating patients.

### Study Endpoint

The study endpoint will correspond to when the patient is discharged from short-stay geriatric care. The duration of study participation for each patient will be the duration of their short-stay geriatric care.

### Evaluation Criteria

The primary endpoint is the mean duration of hospitalization in short-stay geriatric care. The secondary endpoints are hospital mortality rate, assessment of health status of elderly patients hospitalized in short-stay geriatric wards using different criteria and their outcomes following discharge, describing and comparing how the different hotlines across France function, analyzing the principal responses provided to doctors using the hotline, and improving awareness.

### Recorded Variables and Source Data

The study data will be collected directly from the questionnaires as the study progresses. For the first questionnaire (context of calls and solutions proposed), filled out by the hotline geriatric specialist (Textbox 1), health and administrative information will be collected for the study. The health data collected will include the identification of both speakers on the call, patient age, reasons for calling, degree of emergency as perceived by the geriatric specialist and the calling doctor (evaluated by simple scale from 0 to 10; 0 being a null degree of urgency and 10 an absolute urgency), and the responses offered by the geriatric specialist. The administrative data will include call duration (in minutes).

This questionnaire will be anonymous with no identifying criteria. It will be filled out every time contact is made via the hotline, irrespective of what responses are offered by the doctor (referrals to short-stay geriatric care or not).

For the second questionnaire, filled out by the geriatric specialist treating the patient during their hospitalization in short-stay geriatric care following referral from the hotline (Textbox 2), the sociodemographic, health, family, living situation, and location information will be collected for the study.

Sociodemographic data will include the first and last name of the patient, Patient Permanent Identification number, and patient age. These data will be anonymized on entry.

The health data collected will be the reason for hospitalization; number of hospitalizations during the past year; polypharmacy (four or more medications at the same time); biological data [17], including albumin and C-reactive protein levels for assessing nutritional status; and Charlson Comorbidity Index calculation using patient history for assessing the comorbidities of our geriatric population [18-20]; evaluation of activities of daily living (ADL) before hospitalization according to the validated ADL (/6) [21] and instrumental ADL scales (/4) [22]; evaluation of cognitive status using the Mini-Mental State Examination score (/30) [23]; and recommended referral after hospital discharge.

We chose the Charlson Comorbidity Index because it is a validated, reproducible, and simple comorbidity score that measures the impact of age (as there is excess mortality according to age), weighting by age from 1 (50-59 years) to 5 (90-99 years). This score is widely used for geriatrics publications.

Family or marital status data will include single or with a partner, and with or without children. The living situation data will be the residence of the patient (own home, independent-living facility, nursing home). Type of living area will be urban, semiurban, or rural.

**Context of calls and solutions proposed.**

**Caller**

- General practitioner
- Hospital physician
- Other

**Hotline responding doctor**

- Assistant physician
- Hospital practitioner

**Reason for calling**

- Advice
- Emergency department hospitalization
- Emergency geriatric department hospitalization
- Consultation request
- Deferred hospitalization

**Patient age (years), mean (SD)**

**Degree of emergency perceived by the hotline doctor, mean (SD)**

**Degree of emergency perceived by the calling doctor, mean (SD)**

**Response**

- Simple advice
  - Medical
  - Social-medical
  - Therapeutic
- Emergency admission
  - To emergency department:
  - No beds in short-stay geriatrics
  - Emergency care required
- Short-stay geriatric care: allocated bed
  - Yes
  - No
- Deferred hospitalization (days), mean (SD)
- Nonhospital ambulance services
- Outpatient care
- Consultation
- Teleconsultation
- Temporary nursing home

**Call duration (minutes), mean (SD)**

**Sociodemographic participant characteristics.**

**Mean age (SD)**

**Sex**

- Male
- Female

**Mean hospitalization duration (SD)**

**Type of living area**

- Urban
- Semiurban
- Rural

**Reason for hospitalization**

- Fall
- Fainting
- Frailty
- Difficulties coping at home
- Pain
- Stroke
- Change in general status
- Confusion or behavioral problems
- Breathing difficulties
- Sepsis
- Other (specify)

**Residence type**

- Own home
- Nursing home
- Independent-living facility

**Family or marital situation**

- With a partner
- Alone no children
- Alone with children
- Other

**Polypharmacy (>4)**

- Yes
- No

**Charlson Comorbidity Index score, mean (SD)**

**Previous autonomy**

- Mean activities of daily living (SD)
- Mean instrumental activities of daily living (SD)
- Mean blood albumin (SD)
- Mean C-reactive protein (SD)

**Cognitive disorder**

- Mean Mini-Mental State Examination score (SD)
- Diagnosed dementia

**Walking difficulties**

- Walking aids
- History of falls

**Outcomes**

- Return home
- Return to nursing home
- Long-term rehabilitation
- Follow-up care then nursing home
- Admission to nursing home
- Admission to independent-living facility
- Death
- Return home with planned help (specify)

### Statistical Analysis

This is a pilot study. Given the need for subgroup analyses and information concerning the activities conducted at the investigating centers participating in this study, approximately 250 patients are required per center (ie, 2000 in total). This goal is attainable in 24 months, which represents the entire duration of the study. Statistical analysis will be done by statisticians. Different measures will be used depending on the desired descriptive statistics. All analyses will be conducted on the entire included population and each investigating center.

Univariate analysis, descriptive analyses of the study population, will involve recorded variables, comparison of subjects included via the hotline according to sex, and type of discharge using parametric or nonparametric tests and according to the distribution of variables (chi-square tests for quantitative variables, Student *t* tests for qualitative variables, test significance set at 5%).

Multivariate analyses will include analyses of mean values (ANOVA), the use of a generalized linear model, the use of a mixed model for taking into account the random effect of choosing seven centers. A multiple-component analysis will be used to identify different patient groups (patient profile analysis).

## Results

The study was funded and peer reviewed by the National School for Social Security Loire department (École Nationale Supérieure de Sécurité Sociale) and the Conference for funders of prevention of autonomy loss for the elderly of the Loire department in November 2017, and obtained institutional review board approval in April 2018 by the committee for the protection of persons of Sud Est V of Grenoble University Hospital Center (registered under 18-CETA-01 No.ID RCB 2018-A00609-46). Data collection started in May 2018. The planned end date for data collection is May 2020. Data analysis will take place in the summer of 2020. First results are expected to be published in late 2020.

## Discussion

This study will enable us to determine whether a geriatric hotline offers effective management of elderly patients. Could planned hospitalizations be the best solution for our elderly? Will this approach lead to less geriatric syndromes [4-10]?

Wargon et al [4] demonstrated that the elderly spend more time in EDs, which is a potential source of dysfunction in the organization care offered by these services and lowers chances of positive outcomes for the patient. Therefore, there is interest in enabling the assessment of geriatric health status to limit visits to EDs as much as possible and improve the care pathways offered to elderly patients. In a retrospective study, Mazière et al [8] demonstrated that elderly admissions to EDs are independently associated with functional decline in terms of daily activities. The findings of these studies indicate that improving interactions between GP care and hospital services could enable more direct referrals of patients to multidisciplinary care in short-stay geriatric wards, consequently improving the functional prognosis of elderly patients and avoiding geriatric syndromes secondary to ED care.

The study has several strengths. It is a multicentric study covering a large patient sample. The diversity of the participating investigating centers (across three different regions) and their populations is also likely to offer diverse responses. Is the time spent in ED consultations greater in the larger centers? Does this lead to more cases of geriatric syndrome? Are there closer, more effective ties between GP clinics and hospitals in areas with a lower population density? Do these areas (with a lower population density) benefit from better understanding and communication of this type of care? The analysis will be conducted across subgroups (town by town) to avoid confusion bias. The overall population will be sourced from different types of regions (ie, rural, semiurban, urban) to reveal whether this factor has an impact on the knowledge of or access to the hotline [24]. The analysis will enable evaluation of GP clinic-hospital interactions [25] according to population centers, and the need for links between these health care areas, to determine the strengths and limitations and to reinforce GP clinic and hospital collaboration with the aim of reducing ED admissions [26].

By analyzing center by center, we will be able to describe each center with the different responses they offer. Ideally, this will help to improve the function of the geriatric hotline for the entire country and pave the way to new advances, especially in the organization of the care path in the long run.

The analysis also will be conducted in two sections. First, the call to the hotline will be analyzed. Did the experience of the doctor responding to the call influence the type of care recommended and the response offered? Does a high level of perceived emergency correlate to hospitalization via the ED? Is the hotline solely used by general medicine doctors? Would it be a good idea to roll out access to the hotline to other hospital specialties? Given the growing issue of population aging, the current geriatric services are not enough to care for all the elderly. This is why specialists are turning more toward other fields for geriatric assessment (eg, oncology, orthopedics). This study will reveal the different responses that can be provided by this tool (the hotline), from therapeutic or medical advice to referrals for hospitalization, ambulance call-outs, consultations, telemedicine, and more.

Second, we will study the profile of patients referred for hospitalization via the hotline to determine the characteristics of those the hotline recommends for admission to improve their health care approach. Does this tool lead to direct hospitalization in short-stay geriatric care for the less-fragile patients who still have some autonomy? Are the motives for admission less serious? We hypothesize that the mean duration of hospitalization will be shorter in cases in which it is planned, which leads to the question of whether the future of hospitalization is influenced by the ways patients are admitted. In their study involving 520 patients, Dijon et al [27] demonstrated that the intrahospital care pathways of geriatric patients referred directly to short-stay geriatric care are shorter and more effective than those experienced by patients who first went through EDs, with significantly shorter mean hospitalizations achieved through direct hotline-referred admissions (11.6 days versus 14.1 days with ED consultation; *P*<.05). Patients admitted to short-stay care via EDs were also the quickest to be rehospitalized in the future.

Another advantage of our study is its innovative character because there are currently very few studies analyzing geriatric hotlines [25]. In addition, it is observational in nature, which enables many types of procedures from across the country to be analyzed, leading to more standardized approaches.

The study does have limitations, notably its nonrandomized nature. There is no comparison because it is a descriptive study. The quality of the responses will be operator-dependent. Eventually, the goal will be to conduct a second study aiming to compare profiles of elderly patients hospitalized in short-stay geriatric wards via EDs versus those referred via a geriatric hotline. Through the first questionnaire, we will analyze the different responses provided by the hotline (eg, call-outs, consultations, telemedicine, day hospital). We will evaluate the number of hospitalizations avoided or deferred in time following the use of the hotline to reduce inappropriate passage through EDs.

This first study will demonstrate if managing patients by means of a geriatric hotline offers the most effective approach and results in shorter mean hospitalization durations and thus fewer complications and geriatric syndromes and fewer trips to EDs. It will also describe and compare the function of different geriatric hotlines across France to improve health care pathways for the elderly and pave the way toward future advances, such as new modalities of patient management, the development of more appropriate responses to this population, and the reduction of inappropriate visits to emergencies, which we know can be deleterious for the elderly.

## Appendix

Multimedia Appendix 1 Peer-reviewer report from Comite de Protection des Personnes Sud est V.

## Abbreviations

ADL: activities of daily living
ED: emergency department
GP: general practitioner
