# Supplementary material for: Impact of Geriatric Hotlines on Health Care Pathways and Health Status in Patients Aged 75 Years and Older: Protocol for a French Multicenter Observational Study
Source: JMIR Res Protoc. 2020 Feb 13;9(2):e15423. doi: 10.2196/15423 (PMC7055780; doi:10.2196/15423)
Supplement: Multimedia Appendix 1 [file resprot_v9i2e15423_app1.docx]

# Réponse pour le CPP à la demande de modification du protocole

**Ref Etude : Description des hotlines gériatriques en France**

**N° ID RCB : 2018-A00609-46**

Promoteur : Centre Technique d’Appui et de Formation des Centres d’Examens de Santé de l’assurance maladie (CETAF) - SAINT-ETIENNE

Investigateur Principal : Pr Régis GONTHIER - CHU de Saint-Etienne

Type Recherche Biomédicale : TYPE 3

**Objet : Demande de compléments d'informations**

Madame,

Le Comité a reçu le 06/03/2018 le protocole intitulé :

***Etude multicentrique descriptive de différentes Hotlines gériatriques sur le territoire français***

***Description du parcours de soins et de l’état de santé des personnes âgées de 75 ans et plus***

***Multicentric descriptive study of various geriatric hotline in France***

qui a été examiné lors de la séance du 4 Avril 2018 et nous souhaitons vous faire part des observations suivantes :

**Dans le protocole**

**Question 1 : Chapitre X - CONSIDERATIONS ETHIQUES ET JURIDIQUES**

**Page 11 « Patient présentant des troubles cognitifs significatifs (MMS < 20/30) : la personne de confiance sera destinataire de l’information et pourra exercer les droits du patient qui l’a désignée, en son nom. Toutefois, le patient devra recevoir l’information ultérieurement, dès lors que son état de santé le permettra »**

**La personne de confiance n’a pas pour mission d’exercer les droits du patient. Cette mention est à retirer.**

**Réponse réalisée :**

Pièce jointe n°7

**Document décrivant : l’origine et la nature des données personnelles recueillies, la justification du recours à celles-ci, le mode de circulation des données, les destinataires des données personnelles traitées, et la durée de conservation des données.**

***

**DONNEES RELATIVES AUX PERSONNES QUI SE PRETENT A L’ETUDE**

**Origine et nature des données personnelles recueillies**

Les données de l’étude seront recueillies directement par voie de questionnaires, au fur et à mesure des visites de l’étude.

Un premier questionnaire sera complété par le médecin gériatre de la Hotline indépendamment de la réponse qu’il apporte (orientation vers un service de court séjour gériatrique ou non).

Il ne comportera que des données anonymes et non identifiantes, et ne fait donc pas l’objet de développements ici.

Un second questionnaire sera complété par le médecin gériatre suivant le patient lors de son hospitalisation en court séjour gériatrique sur orientation de la Hotline.

Les informations recueillies à cette occasion seront les suivantes :

▪ Données d’identification :

▪ Nom et prénom du patient, numéro d’Identification Permanent du Patient (IPP)

Ces données seront anonymisées lors de leur saisie (voir infra, 11.1)

▪ Données d’ordre signalétique :

▪ Tranche d’âge

▪ Données de santé :

▪ Motifs d’hospitalisation, nombre d’hospitalisations dans l’année, évaluation de la situation médicale (polymédication, score de Charlson [15-17]), antécédents médicaux chirurgicaux, comorbidités, ADL (/6) [18] et IADL (/4) [19], MMS (/30) [20], données biologiques [14] : albumine et CRP afin d’évaluer leur statut nutritionnel, orientation en fin de séjour.

▪ Données relatives à la situation familiale

▪ Données relatives aux habitudes de vie :

▪ Lieu de vie du patient

▪ Urbain, semi-urbain ou rural

Les données sources du 2nd questionnaire seront constituées par l’ensemble des informations et résultats d’examens figurant dans le dossier médical des personnes constitué dans le service de court séjour gériatrique.

**Justification du recours à celles-ci**

Les données recueillies seront strictement nécessaires à l’atteinte des objectifs de l’étude.

Le recueil des données d’identification permettra d’organiser d’éventuels contrôles de données.

Le recueil des données d’ordre signalétique et relatives à la situation familiale permettront de caractériser les patients qui se prêtent à l’étude.

Le recueil des données relatives aux habitudes de vie et aux modes de vies permettra d’évaluer l’impact de ces paramètres sur l’utilisation de la hotline.

Enfin, le recueil des données relatives à la santé est justifié par les références bibliographiques ci-après :

[14] livre-blanc-fragilite-2015.pdf

[15] Charlson ME, Pompei P, Ales KL, MacKenzie CR. A new method of classifying prognostic comorbidity in longitudinal studies: development and validation. J Chronic Dis. 1987;40(5):373‑83.

[16] Charlson M, Szatrowski TP, Peterson J, Gold J. Validation of a combined comorbidity index. J Clin Epidemiol. nov 1994;47(11):1245‑51.

[17] Quan H, Li B, Couris CM, Fushimi K, Graham P, Hider P, et al. Updating and validating the Charlson comorbidity index and score for risk adjustment in hospital

discharge abstracts using data from 6 countries. Am J Epidemiol. 15 mars 2011;173(6):676‑82.

[18] Katz S, Ford AB, Moskowitz RW, Jackson BA, Jaffe MW. Studies of Illness in the Aged : The Index of ADL: A Standardized Measure of Biologie and Psychosocial Function. JAMA. 21 sept 1963;185(12):914-9.

[19] Lawton MP, Brody EM. Assessment of older people: self-maintaining and instrumental activities of daily living. The Gerontologist. 1969;9(3):179-86.

[20] Gluhm S, Goldstein J, Loc K, Colt A, Van Liew C, Corey Bloom J. Cognitive performance on the mini-mental state examination and the Montreal Cognitive Assessment Across the Healthy Adult Lifespan. Cogn Behav Neurol Off J Soc Behav Cogn Neurol. mars 2013;26(1):1-5.

**Mode de circulation des données**

Dans chaque Centre participant, le recueil et la saisie des données seront réalisés par un professionnel de santé habilité participant à l’étude, sous la responsabilité de l’investigateur principal.

Le second questionnaire sera anonymisé préalablement à sa saisie (suppression du nom, prénom et N°IPP des participants), au moyen d’un numéro d’inclusion.

La saisie sera effectuée sur le logiciel LimeSurvey.

Chaque Centre n’aura accès qu’aux données qu’il a produites.

Le logiciel LimeSurvey et les données saisies seront hébergé.e.s chez un hébergeur de données de santé agrée. Les flux de données seront chiffrés et une sauvegarde journalière sera effectuée.

Au terme de la période d’inclusion, le personnel habilité du promoteur en charge de l’analyse des données procèdera à une extraction des données de Lime Survey afin d’importer lesdites données dans le logiciel d’analyse statistique.

Une validation des données sera éventuellement réalisée pour l’analyse statistique, et des demandes de correction pourront être émises auprès de l’investigateur principal ou à un membre de son équipe habilité. Tout contact par mail dans ce contexte interviendra au moyen d’une messagerie sécurisée.

Une fois validées, les données jusque-là conservées dans le logiciel Lime Survey seront supprimées.

En tant que de besoin, il est renvoyé au protocole pour les principales mesures organisationnelles et techniques qui seront mises en oeuvre de manière à garantir la sécurité et la confidentialité des données traitées (§ 10.5).

**Destinataires des données personnelles traitées**

Seul le personnel habilité du promoteur en charge de l’analyse et du contrôle des données sera destinataire des données personnelles traitées.

**Durée de conservation des données**

Dans chaque Centre :

A la fin de la période d’inclusion, l’ensemble des documents de l’étude sur support papier (protocole, classeur investigateur, questionnaires, correspondances) sera conservé dans chaque centre jusqu’à la publication définitive des résultats, et au maximum 5 ans après la fin de ladite période d’inclusion.

Dans le service habilité chargé de l’analyse des données, auprès du promoteur de l’étude :

Une fois le rapport final de l’étude réalisé ou publié et, au maximum dans un délai de 5 ans après la fin de la période d’inclusion, les données figurant sur support informatique seront définitivement extraites par le personnel en charge de l’analyse auprès du promoteur puis conservées sur une clé USB chiffrée, elle-même placée dans une armoire fermée à clé dont l’accès sera réservé au personnel habilité du promoteur.

Une fois ces données extraites, elles feront l’objet d’une suppression dans le logiciel Lime Survey.

La durée de conservation est fixée à 5 ans, aux fins de pouvoir, le cas échéant, effectuer des analyses complémentaires pour une finalité compatible avec la finalité initiale du traitement dans les conditions légales et règlementaires qui s’imposent.

**DONNEES RELATIVES AUX PROFESSIONNELS INTERVENANT DANS L’ETUDE**

La réalisation de l’étude et le respect des obligations légales du promoteur supposent la mise en oeuvre d’un traitement de données à caractère personnel relatives aux professionnels de santé intervenant dans l’étude.

**Origine et nature des données personnelles recueillies**

Les données traitées dans ce contexte proviennent des intéressés eux-mêmes.

Elles sont strictement nécessaires aux finalités précitées, et concernent :

- l’identité desdits professionnels (nom, prénom)

- leur titre professionnel,

- leurs coordonnées professionnelles (adresse postale, adresse mail, et numéro de téléphone).

**Justification du recours à celles-ci**

Le suivi des professionnels de santé intervenant dans la recherche ne peut s’opérer qu’au moyen de données personnelles comportant leur identité, leur titre, et leurs coordonnées professionnelles.

**Mode de circulation des données**

Les données ont été adressées par les professionnels concernés par message électronique.

**Destinataires des données personnelles traitées**

Seuls auront accès à ces données : le personnel habilité du promoteur de l’étude, également responsable de ce traitement, l’investigateur coordonnateur responsable de la recherche, les co-investigateurs, et, éventuellement, les personnels d’autorités légalement habilitées.

**Durée de conservation des données**

Les données traitées dans ce contexte seront conservées dans des conditions garantissant leur sécurité jusqu’à la publication du rapport final de l’étude, et au maximum dans un délai de 5 ans après la fin de la période d’inclusion.

**2^ème^ questions : Dans les différents documents fournis, il faut revoir la terminologie :**

**- Le terme foyer logement est à remplacer par résidence autonomie.**

**- le terme données nominatives doit être remplacé par données personnelles.**

**Réponses apportées :**

**Le terme de foyer logement a été remplacé par résidence autonomie dans le texte.**

**Le terme de données nominatives a été remplacé par données personnelles dans le texte.**
